# Waveguide-integrated mid-IR photodetector and all-optical modulator based on interlayer excitons absorption in a WS_2_/HfS_2_ heterostructure

**DOI:** 10.1515/nanoph-2022-0203

**Published:** 2022-08-22

**Authors:** Shahar Edelstein, S. R. K. Chaitanya Indukuri, Noa Mazurski, Uriel Levy

**Affiliations:** Department of Applied Physics, The Center for Nanoscience and Nanotechnology, The Hebrew University, Jerusalem 91904, Israel

**Keywords:** 2D TMDC materials and heterostructures, integrated mid-IR photodetector, interlayer exciton, silicon and integrated photonics

## Abstract

Novel 2D van der Waals semiconductors facilitate the formation of heterostructures and thus support bandgap engineering for atomically thin modern photonic applications. When these heterostructures form a type II band structure, interlayer excitons (ILEs) are formed as a result of the ultrafast charge transfer between the layers. Here, we present for the first time a waveguide-coupled, mid-IR photodetector and modulator based on the ILE absorption. The device consists of a heterostructure of a single layer of tungsten disulfide (WS_2_) and a few layers of hafnium disulfide (HfS_2_) integrated to a silicon waveguide on a sapphire substrate. We measure broadband mid-IR photodetection (3.8–5.5 µm) with responsivity in the order of tens of µA/W and with no significant effect on the waveguide’s transmission. Additionally, we demonstrate waveguide-integrated, mid-IR, all-optical modulation by controlling the ILE population with the interband transition of the individual layers of the heterostructure.

## 1 Introduction

Transition-metal dichalcogenides (TMDCs) are two-dimensional van der Waals materials with properties ranging from metals, through semiconductors, to novel quantum materials such as topological insulators and Weyl semimetals. Most TMDC semiconductors have a direct bandgap in the monolayer limit, rendering them suitable for optoelectronic applications. Moreover, due to the strong spin–orbit interaction in TMDCs, the valence band splits into different valleys, enabling their use for valleytronics devices. These properties, combined with their atomic-scale thickness and their ease of integration to photonic and plasmonic nanostructures, gave rise to a range of integrated optoelectronic devices including photodetectors, modulators, and sources [1–10]. However, due to the typically large bandgap of these materials, devices based on TMDCs are generally limited to operation in the visible and the NIR spectral regime.

The detection of mid-infrared (MIR) light plays an important role in applications such as material sensing and detection. This is due to the fact that a large variety of molecules have natural vibrational transitions in this region. Unfortunately, commercial free-space spectrometers in this spectral region (e.g., FTIR) are bulky and expensive, whereas a chip-scale counterpart could offer an affordable, miniaturized, and easily integrated alternative. To achieve this goal, reliable, CMOS-compatible, waveguide-integrated mid-IR photodetectors and modulators are needed. Chip-scale integration of conventional narrow bandgap semiconductors such as HgCdTe, group III–V, and group II–VI semiconductors, which are traditionally used for free-space mid-IR detection, is challenging, mostly due to the lattice mismatch between Si and these materials. 2D materials, on the other hand, do not require lattice matching and can simply be put directly on silicon. Furthermore, different 2D materials can be stacked together to form complex heterostructures. This flexibility in integration makes them attractive for integrated photonic devices.

The first 2D material proposed and demonstrated for mid-IR detection was graphene [11–13]. However, its zero bandgap results in a large dark current and a low contrast in signal detection. Moreover, due to the low absolute absorption of graphene, approaches such as cavity enhancement, plasmonic enhancement, or a metal-graphene interface are needed to achieve an observable photoresponse at reasonable power levels [14]. The requirements for metal electrodes and high-thickness graphene (few-layers graphite) complicate the on-chip integration of graphene as it leads to absorption and scattering losses to the propagating modes in the waveguide. Another alternative is to use black phosphorus (BP), which has a small bandgap, around 0.33 eV, in bulk form and can be used as a broadband photodetector up to a cut-off wavelength of 4.13 µm [15]. Several reports on waveguide-integrated BP detectors were published, albeit within the 4 µm wavelength limit [16–18]. From a fabrication perspective, BP is environmentally unstable and must be handled in a glove box. Even after encapsulation, there is some degree of degradation over the device’s life span. Furthermore, in these photodetectors, absorption in the MIR is relatively low, and the coupling of light to the photodetector with a grating coupler is required. Therefore, such photodetectors are mostly integrated at the ends of waveguides. Apart from semimetals, on-chip integrated photodetectors for wavelengths above 4 µm were not yet demonstrated. A few low bandgap 2D materials similar to PdSe_2_ were recently studied [19, 20]. However, fabrication difficulties and lack of bandgap tuning in these materials hinder their use in waveguide-integrated photonics, at least so far. An electrically tunable, narrow bandgap, 2D semiconductor, waveguide-integrated photodetector for the mid-IR may enhance the signal-to-noise ratio and solve the aforementioned problem with semimetal-like (graphene or few-layer graphite) photodetectors.

Mid-IR photodetection based on ordinary interband absorption is not possible in individual TMDC semiconductors due to their large bandgap. However, heterostructures made of TMDCs can form a type II band structure and thus allow the formation of interlayer excitons (ILEs), where a photon excites an electron from the valence band of one material to the conduction band of the other. In general, the ILEs’ oscillator strength is too small to be observed directly in absorption measurements. Recently, however, high oscillator strength ILE generation was reported in a WS_2_/HfS_2_ heterostructure, enabling significant absorption of MIR light. Based on this, free-space MIR photodetection was demonstrated [21]. It remained untested, however, whether the oscillator strength of these ILEs is sufficient to detect light from the evanescent field of a photonic waveguide.

Motivated by this challenge and by the need to demonstrate chip-scale MIR photodetection, we demonstrate in this work the first waveguide-integrated photodetector based on ILEs. The photodetector shows broadband absorption in the MIR region, along with a high level of integration and environmental stability. We also show that the device can operate as a waveguide-integrated all-optical modulator in the MIR controlled by a time-dependent visible-light pump. Among the possible applications are on-chip materials sensing, on-chip frequency references, thermal imaging, MIR communication systems, and perhaps even low two-photon absorption communication via silicon photonics for quantum systems.

## 2 ILE absorption

**Figure 1:**
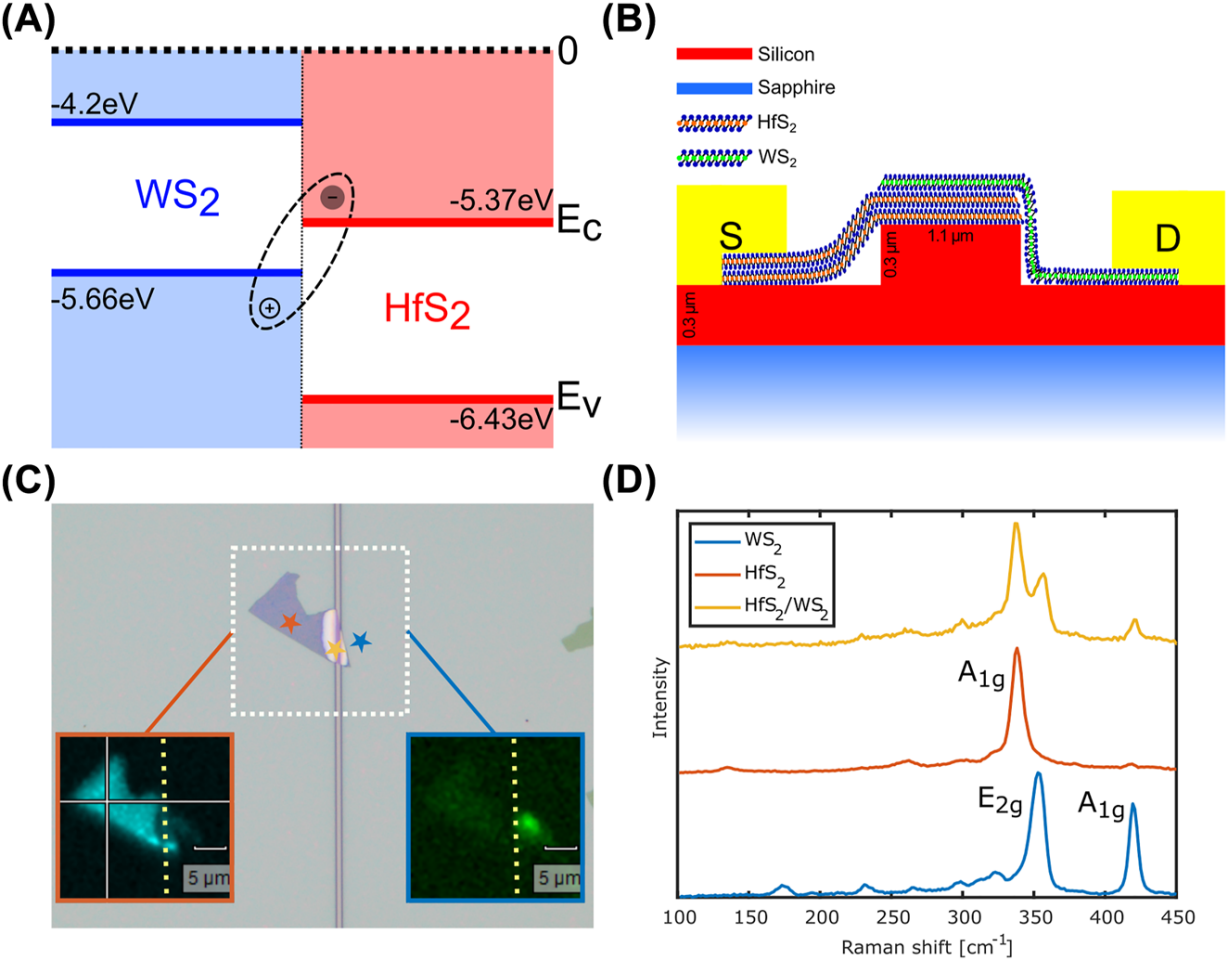
Device structure. (A) Heterostructure band diagram, the energy values of the band edges are relative to the vacuum level and taken from [23]. (B) Schematic description of the device. (C) Optical image of the device without the electrical contacts; the WS_2_ monolayer is not visible. The Blue (bottom right) and red (bottom left) insets show the measured Raman signal from the WS_2_ and the HfS_2_ regions respectively. The dashed yellow lines in these insets represent the location of the waveguide. (D) Raman spectra of the individual layers and the heterostructure; the curves are shifted for clarity.

Figure 1A illustrates the band structure of both parent materials before they are put in contact. When in contact, WS_2_ and HfS_2_ form a type II band structure. The choice of WS_2_ (direct bandgap semiconductor) and HfS_2_ (indirect bandgap semiconductor) is owed to the deep conduction band minimum of HfS_2_, which together with the valence band maximum of WS_2_ forms a small interlayer bandgap of around 0.2 eV. In this heterostructure, the ultrafast charge transfer between the layers enables the formation of space-indirect ILEs with high binding energy (electrons and holes reside in different layers of the heterostructure). However, these space-indirect ILEs are difficult to generate through direct photon absorption, which limits ILE-based photodetectors. Yet, one can improve direct ILE absorption by three different mechanisms. The first is by increasing the spatial wavefunction overlap between electrons and holes in the individual layers. The second is through the conservation of momentum of impinging ILE bandgap energy photons with phonons. This can be achieved by, for example, increasing the density of optical phonon states through temperature. The third is to enhance the interlayer exciton oscillator strength by spatial exciton trapping in the interface between the two layers of the heterostructure.

In common TMDC heterostructures, the ILE oscillator strength is significantly lower than the exciton oscillator strength, *f*_ILE_ ∼ 10^−2^ *f*_exc_, where *f*_ILE_ and *f*_exc_ are the oscillator strengths of ILEs and excitons in the TMDC, respectively [22]. However, by trapping excitons in the interface, one can achieve ILE oscillator strength comparable to *f*_exc_, and these large *f*_ILE_ ILEs can be directly accessed by photons. To increase the spatial wavefunction overlap between electrons and holes, one requires a large hybridization of the wavefunctions of electrons and holes in the heterobilayer. In the case of a WS_2_ and HfS_2_ heterostructure, it was recently reported through ab initio discrete Fourier transform calculations that the hole wavefunction is hybridized between the two layers at the gamma point in the reciprocal space, and spatially indirect ILE is formed between the electron wavefunction in the HfS_2_ conduction band (M point) and the hole wavefunction in the WS_2_ valence band (gamma point) [21]. These HfS_2_/WS_2_ ILEs are momentum-space indirect. Consequently, although the low-energy MIR light cannot excite an electron from the valence band of either of the parent materials to its conduction bands due to their large bandgap, it can excite an electron from the valence band of the WS_2_ to the conduction band of the HfS_2_. The electron-hole pair generated in this process is a wavefunction-hybridized ILE with high oscillator strength and is responsible for the photodetection in this structure.

## 3 Design and fabrication

Figure 1B shows a schematic cross section of the device. A few-millimeter long silicon waveguide is etched on top of a sapphire substrate and terminated with grating couplers on both ends. Sapphire is transparent up to ∼6 µm wavelength and is therefore more suitable as a substrate for MIR devices than the conventional SiO_2_ which is limited to ∼3.5 µm. Si-on-sapphire waveguides can operate in the 1–6 µm range, which is the most significant spectral range for molecular vibrational absorption detection. A few layers of HfS_2_ and a single layer of WS_2_ are polymer dry transferred to the chip with an overlapping region on top of the waveguide. The device is then annealed in a vacuum furnace for 2 h at a temperature of 250 degrees Celsius to establish a conformal contact between the two layers. Chromium gold pads are evaporated on top of the HfS_2_ and WS_2_ layers to form the photodetector contacts.

Optical image and Raman area scans are presented in Figure 1C. The WS_2_ monolayer is not visible in the optical image due to its low contrast with the silicon-on-sapphire surface, but it is clearly visible in the Raman area scan with the blue frame that shows the local intensity of a WS_2_ Raman peak. The area scan with the red frame shows the local intensity of the HfS_2_ Raman peak. The dashed yellow lines in both scans indicate the location of the waveguide. The three stars on the optical image indicate the locations at which the spectral Raman scans in Figure 1D were taken. The Raman data from the area and spectral scans verify the existence of a junction consisting of the two materials on top of the waveguide.

**Figure 2:**
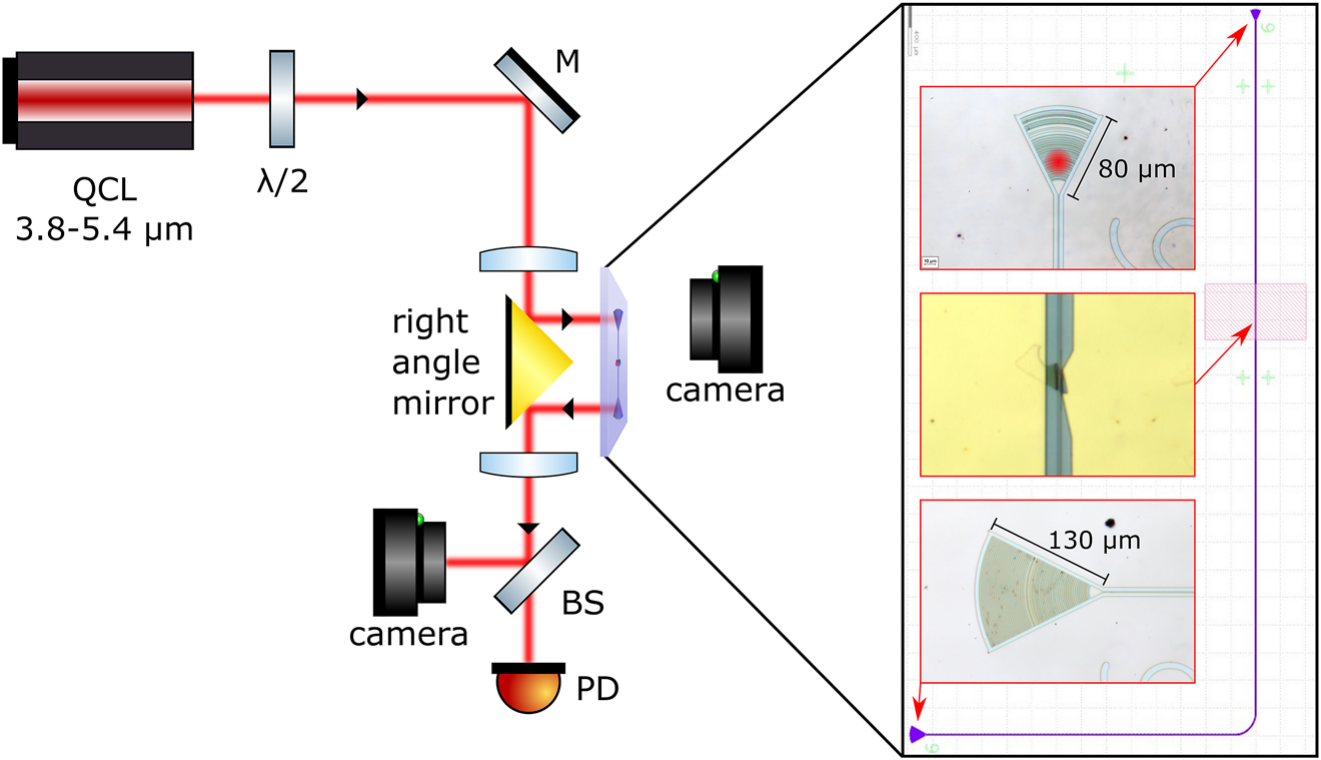
Experimental setup. The inset shows an overview of the waveguide layout and optical images of the grating couplers and the device. The red spot in the top optical image represents the size of the input beam. QCL, quantum cascade laser; *λ*/2, half-wave plate; M, mirror; BS, beam-splitter; and PD, photodetector.

The experimental setup is described schematically in Figure 2. Light from a quantum cascade laser (Daylight Solutions MIRcat-1400) is focused by an 18 mm ZnSe objective lens (Innovation Photonics) to a diameter of ∼25 µm and reflected by a right-angle gold mirror. It is coupled to the input grating coupler of the waveguide from the backside of the chip, through the sapphire substrate. The light is linearly polarized, and a half-wave plate is used to rotate the polarization angle to match the TE polarization of the waveguide mode. To help with the initial alignment of the beam with the grating coupler, a visible laser beam is collinearly aligned with the MIR beam. A visible camera is placed in front of the chip to detect the location of the visible laser spot relative to the input grating coupler. The chip is held by a five-axes stage, and the input grating coupler is brought to the focus of the objective lens. The output grating coupler is located on the far side of the chip, in front of the second facet of the right-angle mirror, so that the output light is sent through a second lens to a commercial mercury–cadmium–telluride (MCT) photodetector (Vigo System). A second visible camera is coaligned with the MCT photodetector to facilitate the alignment of the photodetector with the output grating coupler. The device’s contacts are wire-bonded to a printed circuit board and connected to a source measure unit (Keysight Technologies). The grating couplers have a nonuniform structure and were designed and optimized using 2D FDTD simulations (Lumerical FDTD Solutions) using an algorithm described in [24]. The input coupler was optimized for coupling efficiency from an incoming focused Gaussian beam, and the output coupler was optimized for narrow emission angle to facilitate output signal collection from a distance. The nonuniform design allows for a right angle of incidence of the input beam with a high coupling efficiency. More details of the design of the grating couplers are given in the supplementary information. The overall transmission of the waveguide at the central wavelength (4.34 µm), including input and output coupling and propagation losses, is ∼1.2%.

The magnified insets in the figure show optical images of the input and the output grating couplers and an optical image of a typical device. The red spot on the input grating coupler represents the spot size of the focused input beam. The distance between the two grating couplers is around 1 cm with a distance of a few millimeters from each of the grating couplers to the active photodetector device. The large distance removes any stray light coupling between the couplers and the photodetector such that all the light that is coupled to the input grating coupler is either absorbed by the 2D materials or reaches the output coupler.

## 4 Results and discussion

**Figure 3:**
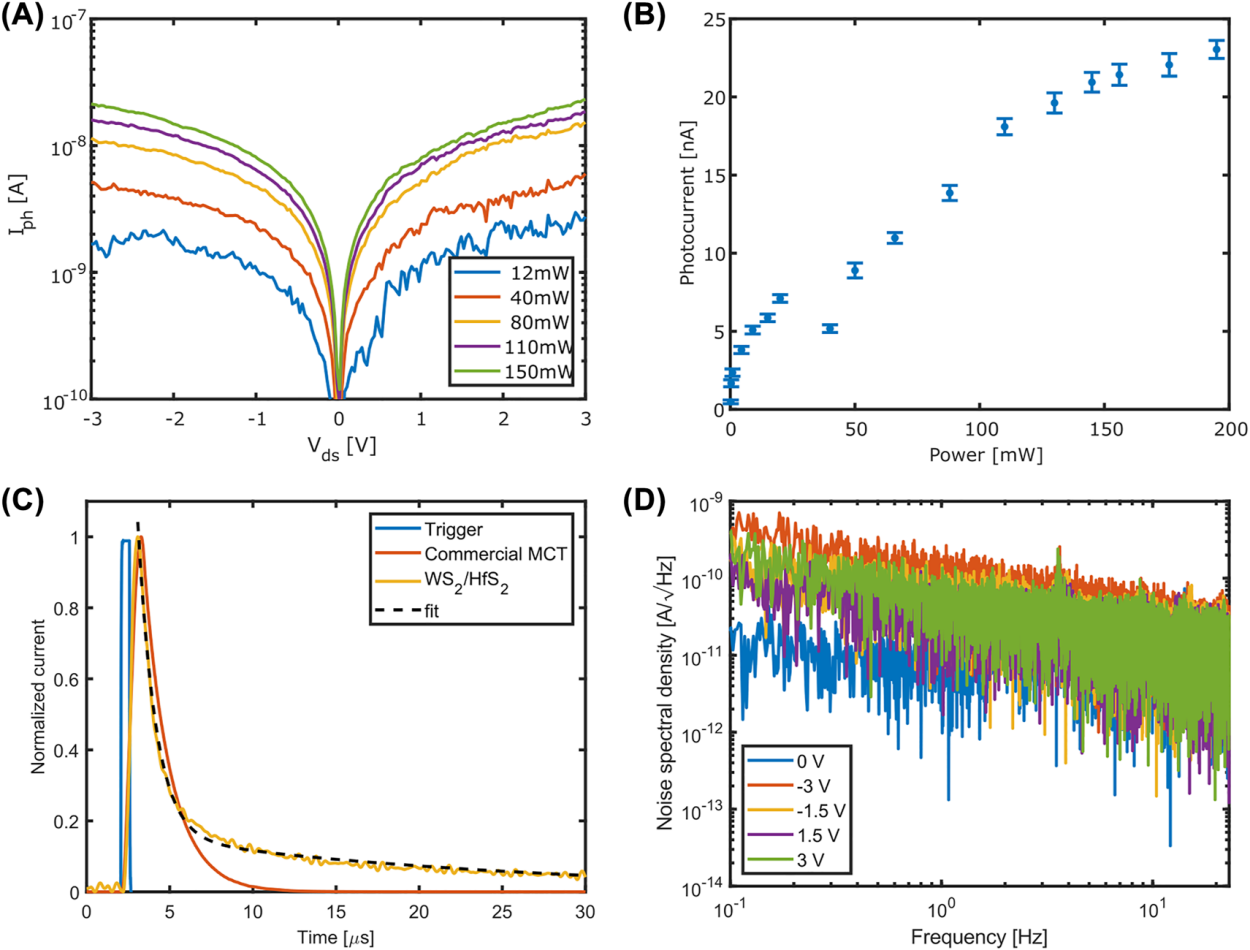
Photocurrent and time response of a WS_2_/HfS_2_ photodetector. (A) Measured *I*–*V* curves for different optical powers at 4.34 µm wavelength. (B) Measured photocurrent as a function of optical input power. (C) Measured time response of the device compared to a commercial MCT photodetector. The trigger curve shows the duration of the laser pulse. (D) Measured noise spectral density as a function of frequency for different bias voltages.

Figure 3A shows the IV characteristics of the waveguide-integrated photodetector measured at a wavelength of 4.34 μm at different laser powers. The photocurrent is defined as *I*_ph_ = *I*_total_ − *I*_dark_ and *V*_ds_ is the applied source-drain voltage. The total current curves are available in the supplementary material. The specified laser power is measured before the focusing lens. From measuring the optical power at the output of the waveguide, we estimate the power inside the waveguide to be roughly one order of magnitude lower. The nonrectification in the photocurrent as a function of *V*_ds_ indicates that our heterostructure behaves similar to a photoconductor, and that an Ohmic contact is formed between the 2D materials and their electrodes.

Figure 3B shows the photocurrent at *V*_ds_ = 3 V as a function of the laser power. We notice a clear saturation effect. At low intensities, the charge carriers’ photogeneration efficiency is linearly proportional to the photon flux, whereas at higher intensities it deviates from linearity. This behavior is similar to the power-dependent interband absorption saturation observed in individual TMDC materials [15, 25]. In the low-power region, in the linear region of the power dependence, we measure maximum responsivity figures of ∼50 μA/W. The lowest input power that generated a measurable current was ∼200 µW. Similar photocurrent characteristics observed over three more devices (supplementary information) indicate the robustness and reproducibility of the interlayer-exciton-based photodetection in the waveguide integrated HfS_2_/WS_2_ heterostructure. Both possible stacking orders (HfS_2_–WS_2_–Si and WS_2_–HfS_2_–Si) were tested, and they demonstrated similar performance (details in supplementary information S1). Although a greater number of each configuration is needed to perform a comparative study, we believe that the low mid-IR absorption of each of the parent materials and the nanometer thickness of the layers limit the stacking order importance.

As mentioned, these ILEs are momentum-space-indirect and thus, for their photogeneration, additional lattice phonons are required for momentum conservation. With temperature, one can control the lattice phonon density of states and thereby control the ILEs’ generation rate. We experimentally observed the enhanced photoresponse when increasing the temperature of our devices. More details regarding the temperature-dependent photoresponse measurements and noise measurements are given in the supplementary material.

The photodetector’s time response is presented in Figure 3C, and compared to a commercial MCT detector. The response was measured using a current amplifier with a rise/fall time of 900 ns (10–90%). The fall time is fitted with a biexponential decay, *i*(*t*) = *A*_1_ exp(−*t*/*τ*_1_) + *A*_2_ exp(−*t*/*τ*_2_), with *τ*_1_ = 1.07 μs and *τ*_2_ = 22.1 μs. The longer time constant corresponds to the device RC time constant, consistent with CV measurements of the device (supplementary material). This biexponential decay is consistent with all other devices. The time response does not depend on the polarity of the bias voltage. Based on the responsivity and noise spectral density measurements (in Figure 3D), we estimate the noise-equivalent power (NEP) to be ∼1 μW/Hz^1/2^, where the power refers to the total power of the guided mode and not the power absorbed in the detector. The relatively high NEP is due to heterostructure operation as a photoconductor which results in a large dark current. However, one can reduce the dark current with bandgap engineering, using another 2D TMDC material as a barrier layer [26]. Furthermore, it should be noted that the measured noise spectral density is about three orders of magnitude higher than the shot-noise-limited noise spectral density. Although the reasons for this discrepancy are a subject for further investigation, we do believe that the NEP can be significantly improved by operating next to the shot noise limited regime.

For both the responsivity and the NEP calculations, we have taken into account the total power in the waveguide, as opposed to the fraction of the mode that overlaps with the detector cross section. This choice provides us with a more conservative estimation for these figures and may be more useful as a full-system characterization. However, our waveguide geometry is not optimal, and its design greatly affects the performance of the detector. Therefore, to fully characterize the photodetector, it is useful to examine the mode overlap of the heterostructure. Using a mode solver (Lumerical), we found that ∼0.33% of the mode power overlaps with the 2D materials (more details and mode profile can be found in the supplementary material). This figure indicates room for improvement in the waveguide geometry.

**Figure 4:**
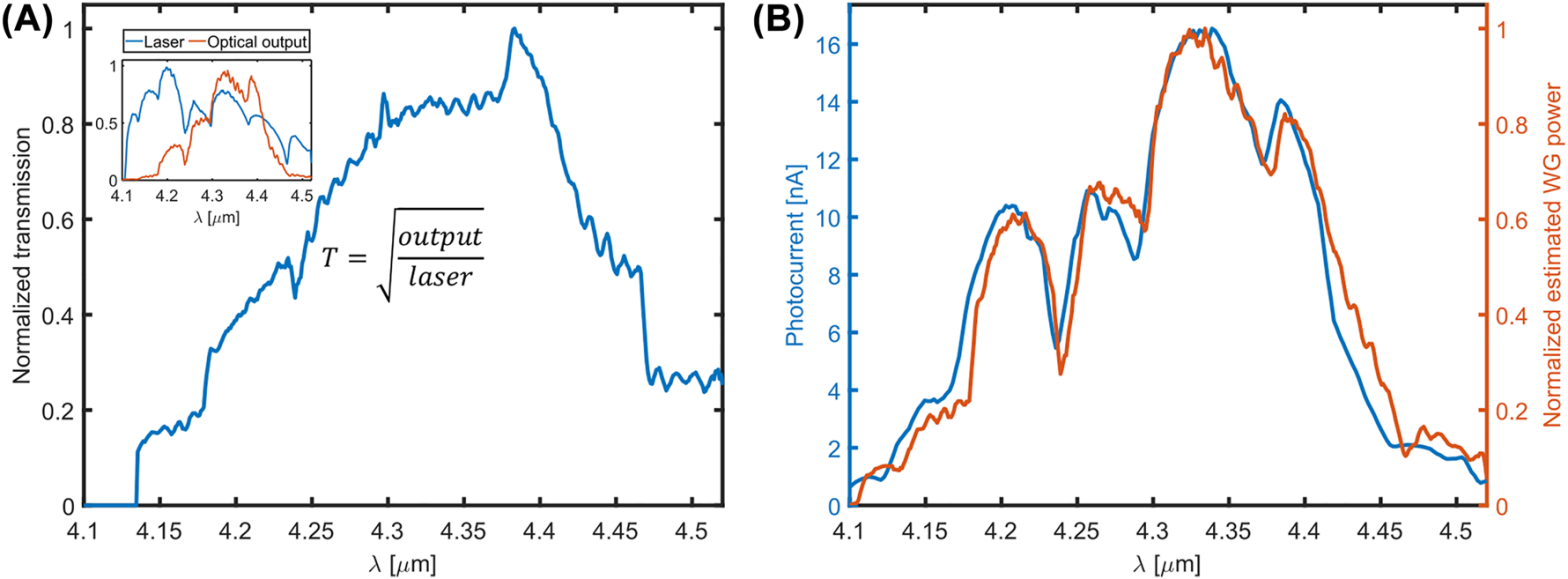
Spectral response of the photodetector. (A) Estimated transmission spectrum of the input grating coupler, equal to the square root of the total transmission of the waveguide. Inset: input and output spectra. (B) Spectra of the photocurrent and the estimated optical power propagating in the waveguide.

To verify whether the photocurrent is a result of the light inside the waveguide and not of a stray reflection from the setup, we checked the spectral correlation between the photocurrent and the guided power. To estimate the guided power, we estimated the transmission spectrum of our input waveguide to be roughly the square root of the measured total transmission of the waveguide (Figure 4A). The total transmission is based on the input and output measurements shown in the inset. The spectra of the estimated guided power and the photocurrent are presented in Figure 4B, and the clear correlation between them verifies that the photocurrent arises from the light in the waveguide. As evident from Figure 4, the spectral response of the device is determined mainly by the transmittance of the grating coupler. Therefore, to isolate the spectral response of the WS_2_/HfS_2_ junction, we used a top illumination scheme and received a broadband photoresponse in the range of 3.8–5.5 µm, limited only by our laser scanning range. In this range, we did not observe any peak or segment of the expected Gaussian-like curve, suggesting that the actual spectral response of the heterostructure is significantly broader, in agreement with the measurements presented in [21].

**Figure 5:**
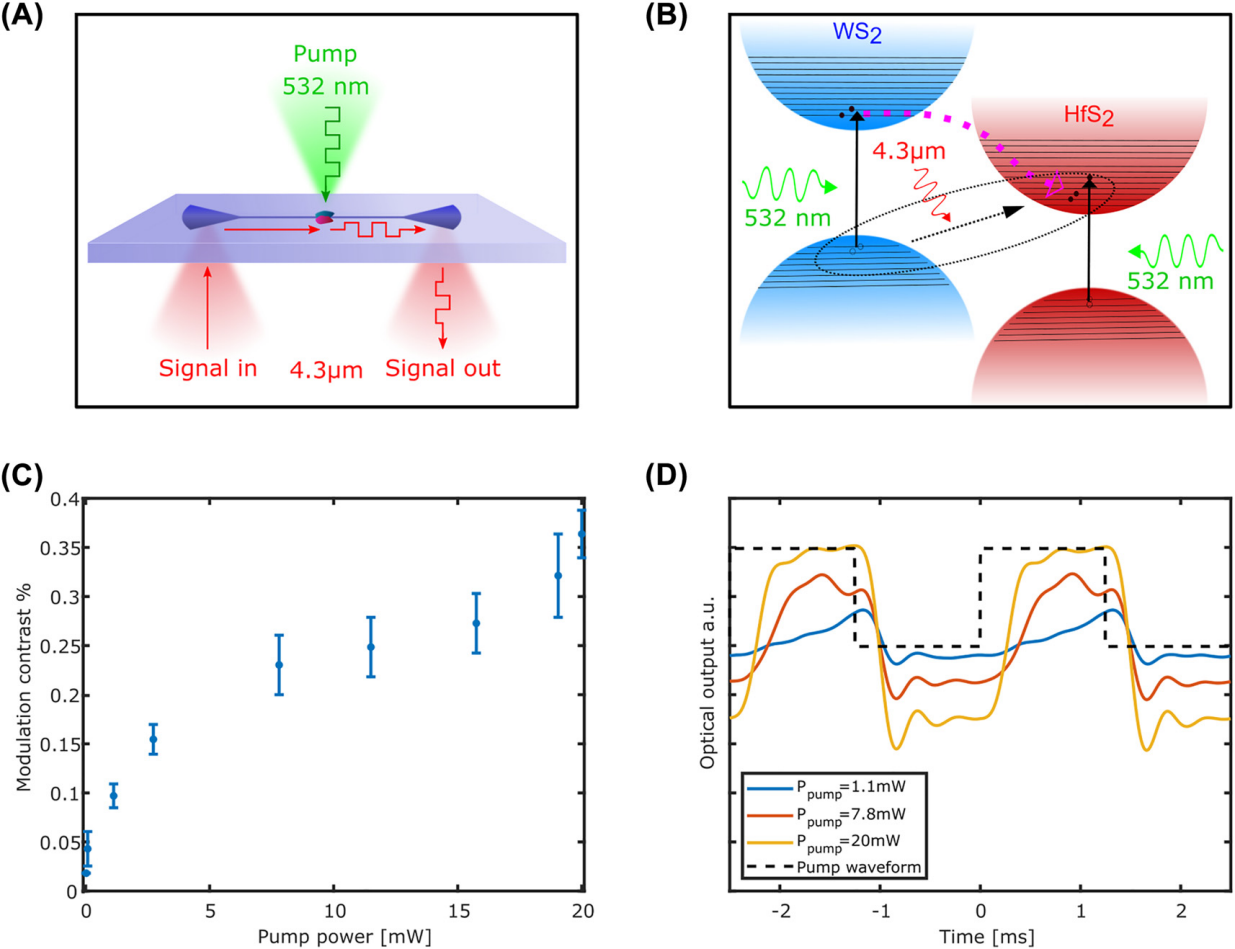
All-optical modulation. (A) All-optical modulation schematics. (B) Energy band diagram for all-optical modulation. (C) Modulation contrast as a function of the pump power. (D) MIR output signal waveforms for different pump powers (only the AC component of the waveform is presented).

Apart from photodetection, with the same device, we demonstrated for the first time all-optical modulation based on ILEs in the MIR. In this setup, depicted in Figure 5A, we modulate the transmission of the MIR light propagating through the waveguide by a 532 nm laser light focused on top of the device. The laser is focused on the junction with an objective lens (50×) and is switched on and off to create a square wave with a frequency of 400 Hz. Figure 5B shows the simplified energy band diagram of WS_2_/HfS_2_ heterostructure. The green light (green arrow) photons have higher energy than the bandgap of each of the parent materials of the heterostructure and are therefore absorbed in both. This absorption generates interband excitons in both HfS_2_ and WS_2_ layers, as indicated by the black arrows. The electrons generated in the WS_2_ layer by the 532 nm excitation hop to the conduction band of the HfS_2_ and fill more electron energy levels in the conduction band of the HfS_2_. This reduces the probability of ILEs generation due to the unavailability of vacant electron energy states in the HfS_2_ conduction band and thus reduces the interlayer exciton absorption (black arrow). This results in lower absorption and consequently in a higher MIR transmission through the waveguide. Figure 5C shows the modulation contrast as a function of the pump power. The modulation contrast is calculated as the ratio of the peak-to-peak voltage of the output signal normalized to its value when the pump is off. Clearly, the modulation contrast is limited by the maximal absorption in the “off” state (i.e., the state where the pump is not applied). To achieve maximal contrast, one needs to use a sample that is long enough to provide full absorption, or alternatively to enhance the absorption by using approaches such as photonic cavities or plasmonic nanoantennas. From the apparent saturation in the curve, we can deduce that, at these power levels, the junction is practically transparent to the MIR signal. Therefore, the maximum modulation contrast gives us an experimental approximation of the fraction of the guided light that is absorbed in the detector without modulation. A more straightforward approach, such as measuring the transmission of the same waveguide before and after the transfer of the device, would not be meaningful since variations in coupling efficiencies are more significant than the losses caused by the WS_2_/HfS_2_ junction. Figure 5D shows the modulated MIR output signal of the waveguide for three different pump powers. When the pump (black dotted line) is on, the MIR transmittance increases, and when it is off the transmittance reduces. In this experiment, we used an MIR wavelength of 4.3 µm. However, due to the broad interlayer absorption spectrum, one can achieve with this heterostructure an all-optical modulation over a spectral range of 3–10 µm. The photoresponse of the heterostructure device was not altered by the high visible pump intensities used for the all-optical modulation experiment.

It is worth mentioning that the responsivity of our waveguide integrated photodetector is of the order of ∼50 μA/W, which is low when compared to other reported interlayer-absorption-based photodetectors made of TMDC materials in free space configuration. Indeed, a planar HfS_2_/WS_2_ device was reported recently with significantly higher responsivities [21]. It is well-known that the oscillator strength of ILEs is two to three orders of magnitude smaller than that of interband excitons within each layer, resulting in an ILE photoresponse smaller on the same scale compared to direct band absorption photoresponse in individual TMDCs. However, in the case of a planar HfS_2_/WS_2_ heterostructure, the band bending at the interface of WS_2_ and HfS_2_ creates a potential trap for ILEs and as a result, ILE densities are comparable with exciton densities in the individual layers. These high-trapped ILE densities result in a large *f*_ILE_ when compared to *f*_exc_. In a waveguide-integrated HfS_2_/WS_2_ heterostructure photodetector, due to the strain-dependent bandgap modification, the band bending between HfS_2_ and WS_2_ is small compared to planar devices. This reduces the oscillator strength of the ILEs and the corresponding photoresponse in the waveguide-integrated photodetector. This can be overcome by the planarization of the waveguides by local oxidation of silicon to form silicon waveguides on sapphire, as used in conventional SOI waveguides technology [27, 28]. Another reason for low responsivity is the low absorption due to the small overlap between the optical mode and the HfS_2_/WS_2_ heterostructure. The overlap can be improved by further optimizing the waveguide structure. Future work will consider new strategies to enhance the absorption of ILEs. Examples for such approaches include phonon density of state engineering, enhancement of the oscillator strength, external stimulus, and plasmonic-photonic-based nanostructures integrated into the waveguide.

Another aspect that can be further improved is the coupling efficiency from free space into the chip. Our current grating couplers provide coupling efficiency slightly higher than 10%. This can be further improved by implementing new design methods like inverse design or by using subwavelength gratings. Alternatively, coupling the light at a small angle may improve the coupling as well. Some of these approaches will be addressed in the future.

## 5 Conclusion

In conclusion, we have demonstrated, for the first time, a waveguide-integrated, ILE-based, MIR photodetector with a responsivity of around 50 μA/W and a spectral operating region beyond the conventional BP cut-off wavelengths of 4 µm. Moreover, the electrically tunable bandgaps of ILE-based devices enable broadband, electrically controlled, IR photodetectors on-chip [21]. Additionally, we have demonstrated waveguide-integrated, all-optical modulation in MIR wavelengths. The latter can be used for applications such as the conversion of information from visible wavelengths to the MIR wavelength regime. We also indicated future directions for enhancing the photoresponsivity of these ILE-based MIR photodetectors.

## Supplementary Material

Supplementary Material Details
