# Supplementary material for: Waveguide-integrated mid-IR photodetector and all-optical modulator based on interlayer excitons absorption in a WS2/HfS2 heterostructure
Source: Nanophotonics. 2022 Aug 22;11(19):4337–45. doi: 10.1515/nanoph-2022-0203 (PMC11502041; doi:10.1515/nanoph-2022-0203)
Supplement: Supplementary file 1 — Supplementary Material Details [file j_nanoph-2022-0203_suppl.docx]

Supplementary infromation

Waveguide-Integrated Mid-IR Photodetector and All-Optical Modulator Based on Interlayer Excitons Absorption in a WS_2_/HfS_2_ Heterostructure

Shahar Edelstein, S.R.K. Chaitanya Indukuri, Noa Mazurski, and Uriel Levy^*^

*Department of Applied Physics, The Center for Nanoscience and Nanotechnology, The Hebrew University, Jerusalem 91904, Israel*

** ulevy@mail.huji.ac.il*

This Supplementary information contains:

Figure S1: Optical images of all devices.

Figure S2: IV characteristics and photocurrent response of all devices.

Figure S3: Photocurrent as a function of power with saturation model fit.

Figure S4: Effect of temperature on photocurrent response in interlayer exciton devices.

Figure S5: Simulated transmission spectra of the input and output grating couplers.

Figure S6: FDTD simulated and measured transmission spectrum through both input and output grating couplers.

Figure S7: Photocurrent spectral response of the device under top illumination.

Figure S8: CV measurement of the heterostructure device.

Figure S9: Power mode profile of the waveguide with the heterostructure.

1. **Optical images**

**
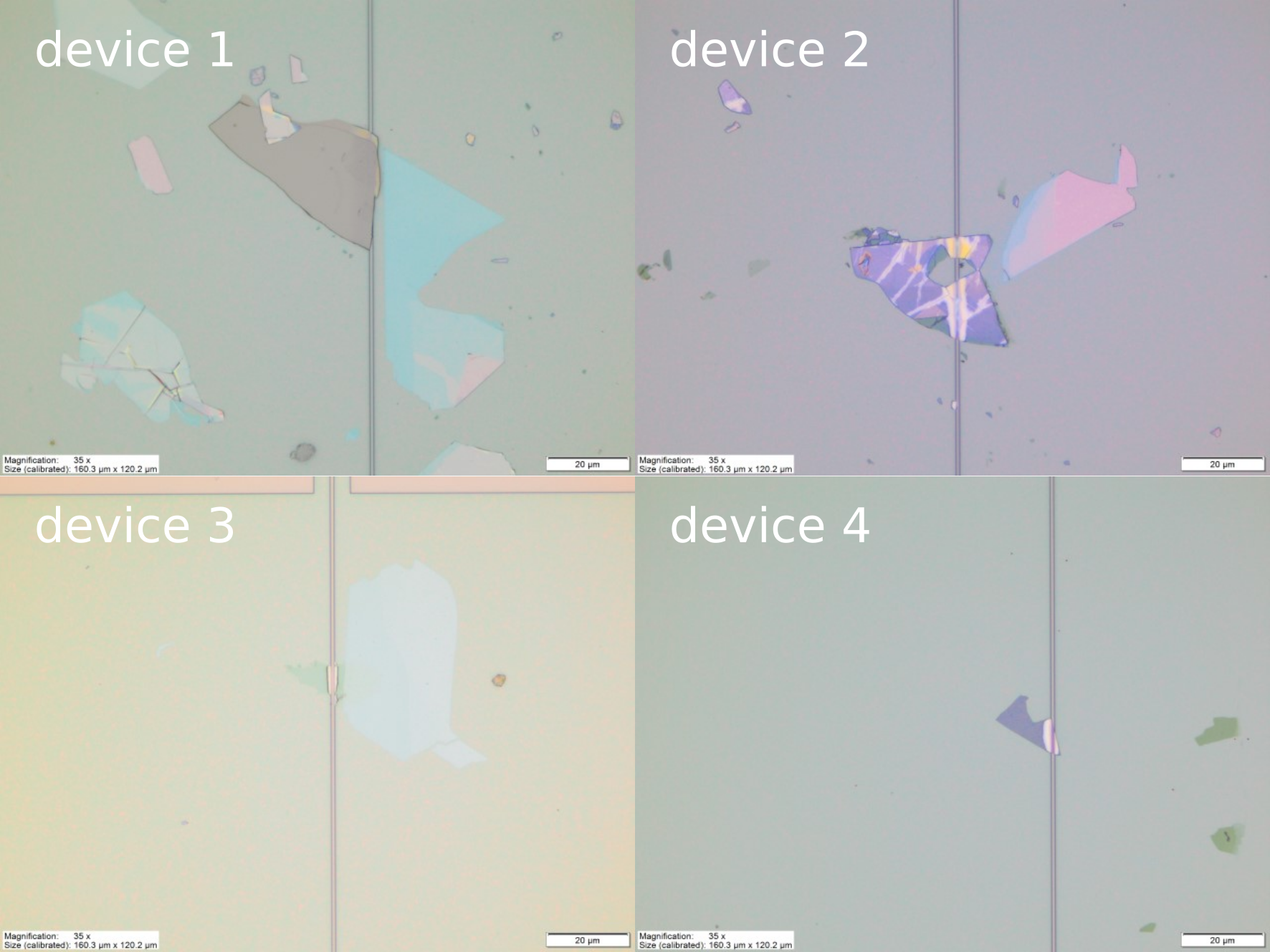
**

Figure S1: Optical images of all devices. The stacking order of devices 1 and 4: HfS2-WS2-Si. The stacking order of devices 2 and 3: WS2-HfS2-Si.

1. **IV curves**


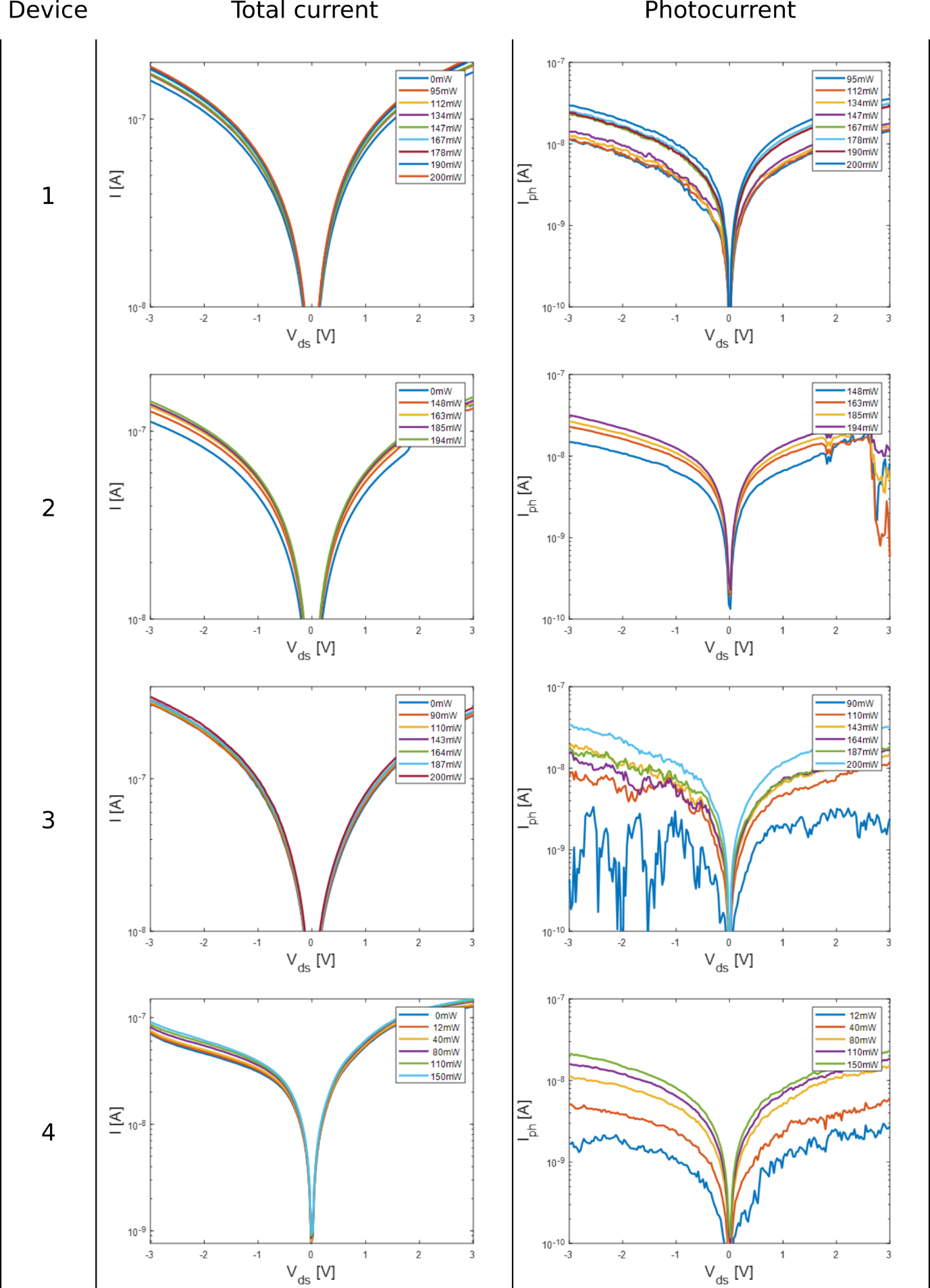


Figure S2: IV characteristics and photocurrent response of all devices.

1. **Photocurrent fit**

**
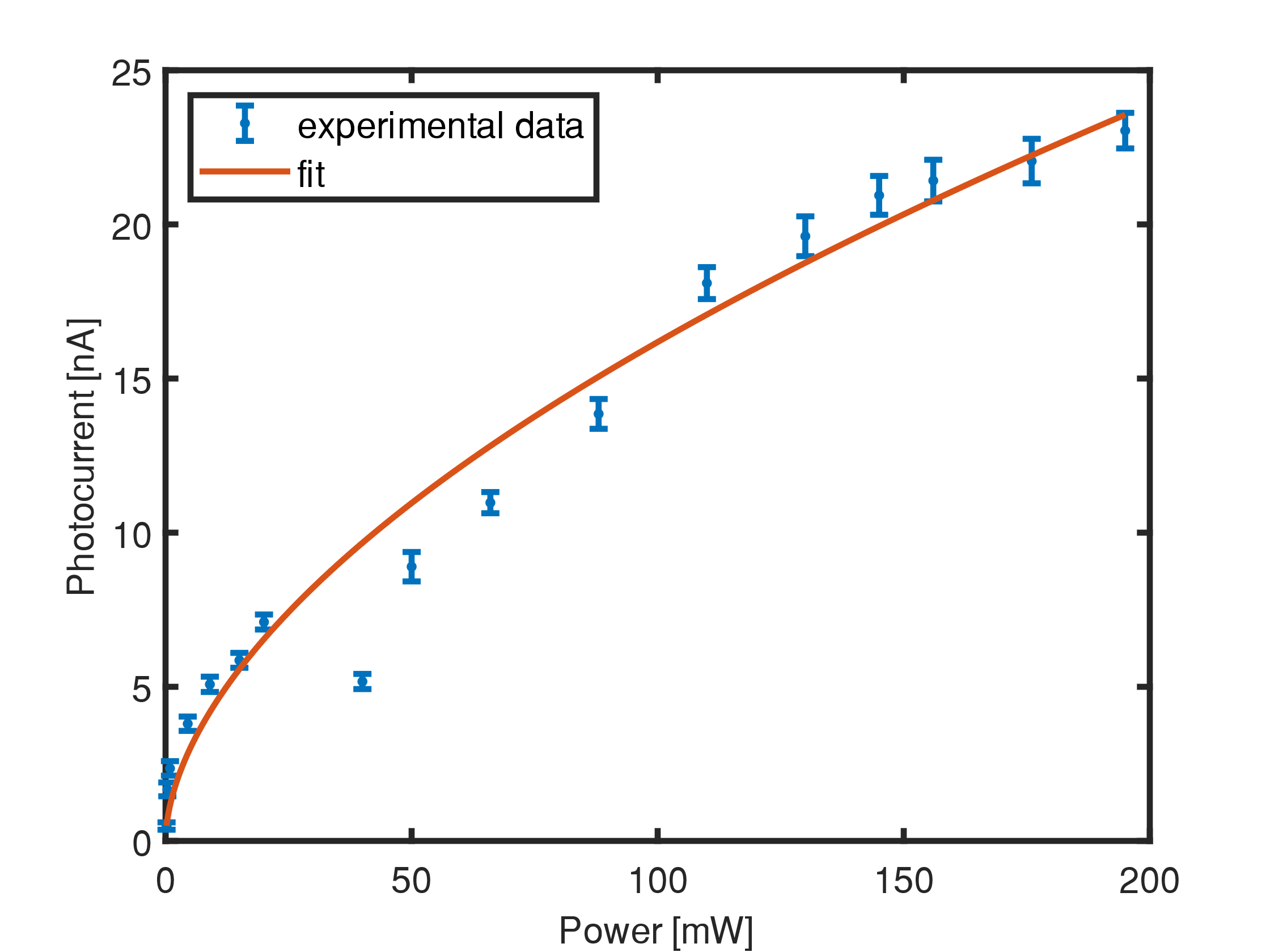
**

Figure S3: Photocurrent as a function of power with saturation model fit. The fitting function is $I_{ph}=AP^{\alpha}$ with parameters $A=1.215\times{10}^{-9}, \alpha=0.562$, consistent with previous observations of power dependence of the photoresponse in TMDC photodetectors  [1].

1. **Temperature dependence**


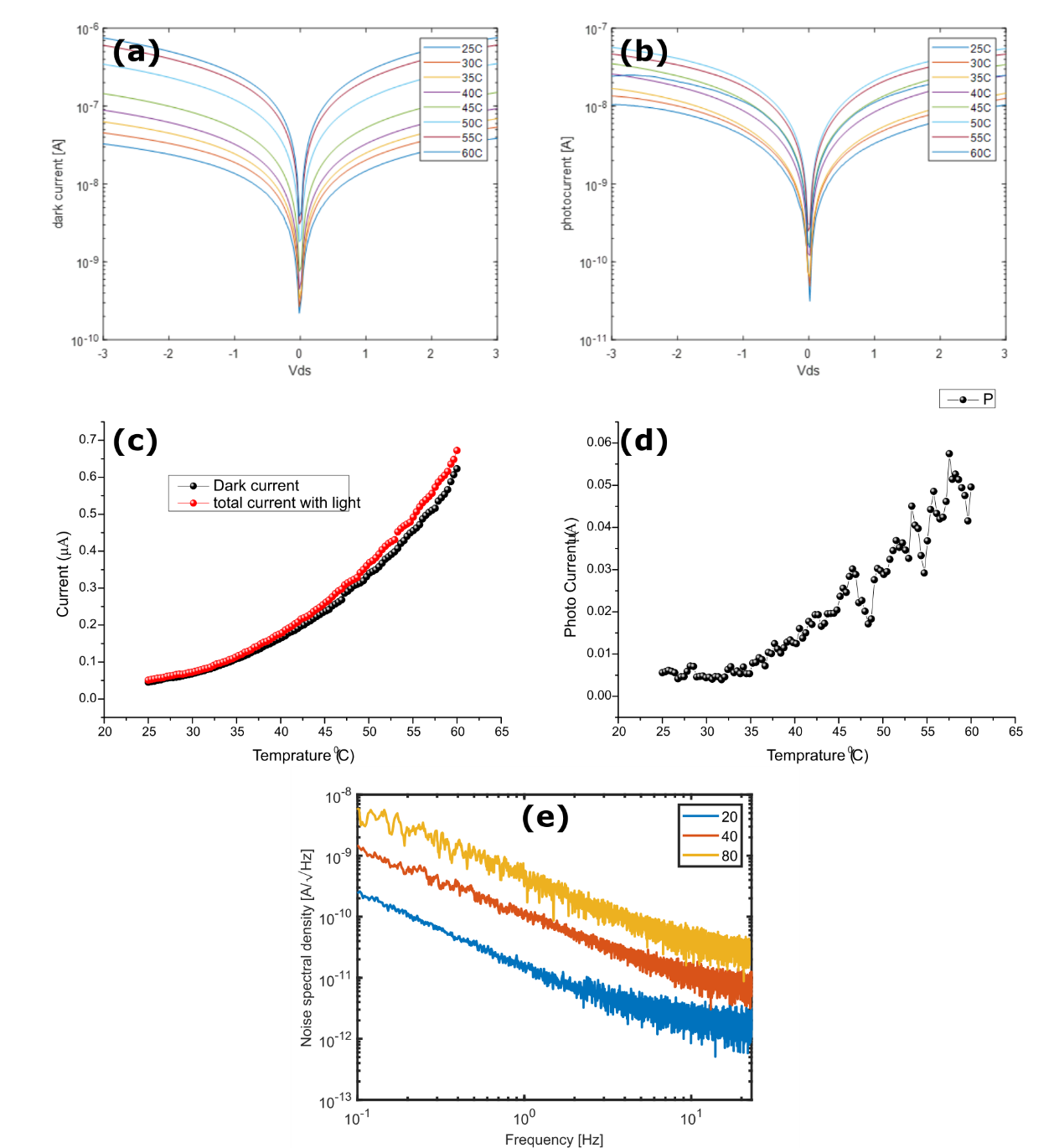


Figure S4: Effect of temperature on photocurrent response in interlayer exciton devices. (a) Dark current IV curves as a function of temperature. (b) IV curves under for input power of 100 mW as a function of temperature. (c) Peak dark and total currents from (a) and (b) as a function of temperature. (d) Photocurrent as a function of temperature. (e) Noise spectral density for temperatures of 20, 40 and 80 degrees centigrade.

1. **Grating coupler transmission**

The grating couplers are nonuniform, meaning that each grating element is free to have, within certain limits, a different period and duty cycle. This approach was chosen to improve the coupling of a right-angle incident beam. Usually, grating couplers are designed for a small incident angle that breaks the symmetry and thus improves the coupling to one direction of the grating coupler. For setup simplicity reasons, we preferred using right-angle incidence, and therefore the symmetry must be broken in the grating coupler itself. were designed using FDTD (Lumerical) and optimized in an iterative process following the principle described in  [2]. First, a uniform grating coupler is designed for the desired wavelength and is used as a basis for the nonuniform optimization. In the next step, each grating element is optimized independently, and by order, for the optimal periodicity and duty cycle. This is done by randomly generating a set number of possible geometries and measuring a figure of merit for each one of them. Then, the geometry with the highest figure of merit, if it improves upon the existing geometry, is chosen for the design. When all grating elements are optimized, the process returns until no significant improvement is obtained. The limits for the possible geometries are dictated by the fabrication technology. In our case, details (ridges or valleys) smaller than 80 nm were not allowed. The figure of merit for the input grating coupler was the transmission (as a fraction of the source power) to the waveguide. For the output coupler, the figure of merit was the total power emitted within an angle of 10 degrees from the perpendicular direction in the far field.

Figure S5: Simulated transmission spectra of the input and output grating couplers

Figure S6: FDTD simulated and measured transmission spectrum through both input and output grating couplers.

1. **Broadband response for top illumination**

Figure S7: Photocurrent spectral response of the device under top illumination (not through the waveguide).

1. **CV measurement**

Figure S8: CV measurement of the heterostructure device.

1. **Mode profile**

**
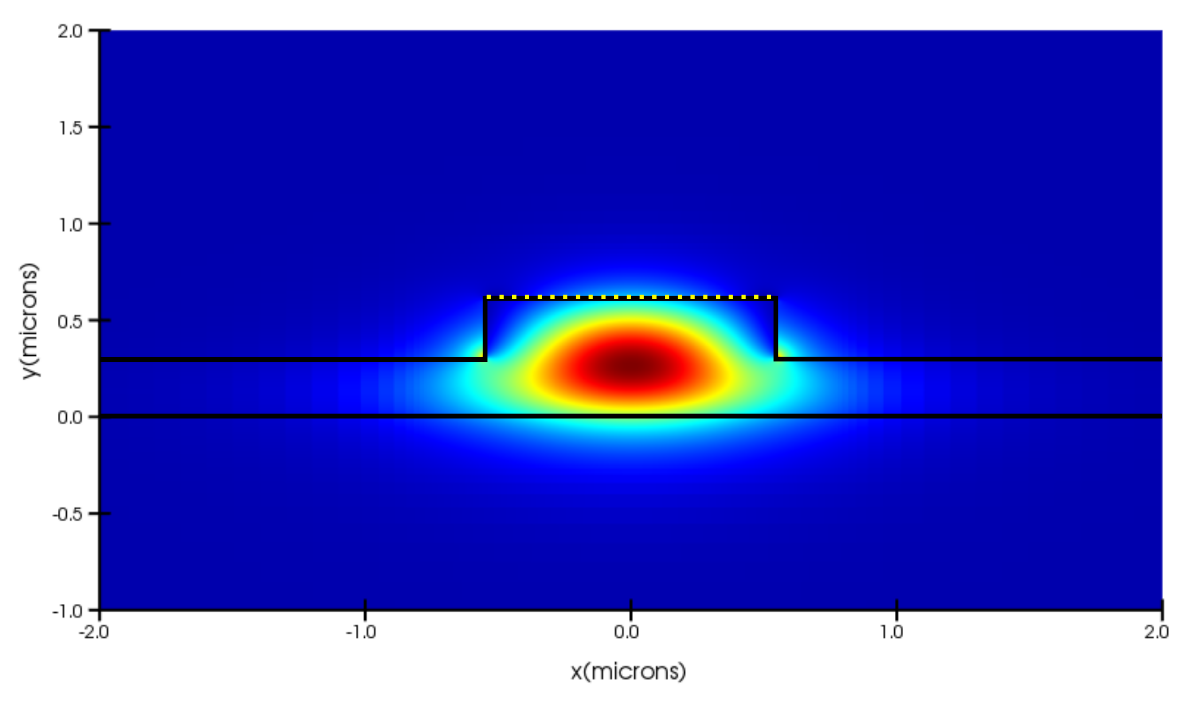
**

Figure S9: Power mode profile of the waveguide with the heterostructure. The black lines indicate the silicon structure outline. The yellow dashed line indicates the location of the 2D materials heterostructure. The wavelength is 4.3 μm. For the simulation we have taken a 10 nm HfS_2_ layer (few atomic layers) and a 0.7 nm WS_2_ layer (monolayer) with refractive indices of 2 and 2.7 respectively [3,4].

References

1. B. Li, L. Huang, M. Zhong, Y. Li, Y. Wang, J. Li, Z. Wei, B. Li, L. Huang, M. Zhong, Y. Li, Y. Wang, J. Li, and Z. Wei, "Direct Vapor Phase Growth and Optoelectronic Application of Large Band Offset SnS2/MoS2 Vertical Bilayer Heterostructures with High Lattice Mismatch," Adv. Electron. Mater. **2**, 1600298 (2016).

2. W. S. Zaoui, A. Kunze, W. Vogel, M. Berroth, J. Butschke, F. Letzkus, and J. Burghartz, "Bridging the gap between optical fibers and silicon photonic integrated circuits," Opt. Express **22**, 1277 (2014).

3. Mohammadreza Razeghizadeh and Mahdi Pourfath, "First principles study on structural, electronic and optical properties of HfS 2(1−x) Se 2x and ZrS 2(1−x) Se 2x ternary alloys," RSC Adv. **12**, 14061–14068 (2022).

4. G. A. Ermolaev, D. I. Yakubovsky, Y. V. Stebunov, A. V. Arsenin, and V. S. Volkov, "Spectral ellipsometry of monolayer transition metal dichalcogenides: Analysis of excitonic peaks in dispersion," J. Vac. Sci. Technol. B, Nanotechnol. Microelectron. Mater. Process. Meas. Phenom. **38**, 014002 (2019).
